# Pneumococcal Bacteremia Presenting as Acute Parotitis and Sepsis

**DOI:** 10.1155/2009/627170

**Published:** 2009-09-10

**Authors:** Ashish Kataria, Alan S. Multz

**Affiliations:** Department of Medicine, Long Island Jewish Medical Center, North Shore-Long Island Jewish Health System, New Hyde Park, NY 11040, USA

## Abstract

We report a case of a 33-year-old female with history of Systemic Lupus Erythematosus (SLE) presenting with acute febrile illness and unilateral parotid gland enlargement progressing to septic shock. The chest imaging showed bilateral multilobar infiltrates and Pneumococci were identified in the blood cultures. The patient was treated with broad-spectrum antibiotics. The underlying imunosupression caused by SLE and long-term steroid treatment could have predisposed this patient to invasive Pneumococcal disease.

## 1. Introduction

Parotitis is usually a polymicrobial infection caused by *Staphylococcal aureus* and anaerobic organisms [1, 2]. It is rare for Pneumococci to seed the parotid gland [3, 4]. However, it is important to recognize the bacterial seeding of the parotid gland and its potential to cause sepsis especially in a predisposed individual.

## 2. Case Presentation

A 33-year-old Indian female with a history of SLE for 5 years presented to an emergency department (ED) after a feeling of neck pain, lump, and fever for 2-day duration. She had been having a mild dry cough for the same duration. The 3–5 cm neck lump was in the left parotid region, and was tender, but not erythematous or fluctuant and was not draining any material. There were bibasilar crackles in the lungs. The abdomen was benign and there were no meningeal signs. Within hours of arrival into the hospital, she became hypotensive and lethargic requiring vasopressor support. The patient was intubated in the intensive care unit (ICU) and started on broad-spectrum antibiotics with Vancomycin, Imipenem, and Mycofungin, in view of her immunocompromised status. The patient had been on oral steroids for the last several years and on infliximab for several months for her lupus nephritis.

## 3. Hospital Course

The patient had a CT scan of neck and chest in the ED. Complete blood count, metabolic panel blood cultures, urine cultures, and urinalysis were sent at the time of the admission.

**Figure 1:**
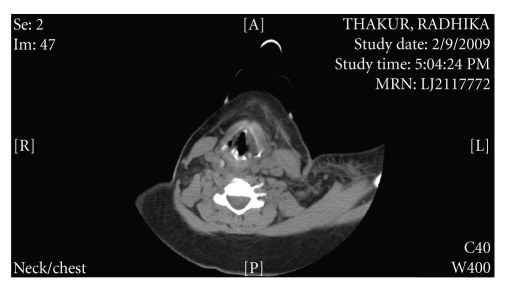
CT Neck showing the abscess.

The CT of neck (Figure 1) showed findings suggestive of left parotitis and inflammation spreading to left pharyngeal mucosa and parapharyngeal space and retropharyngeal space. 

**Figure 2:**
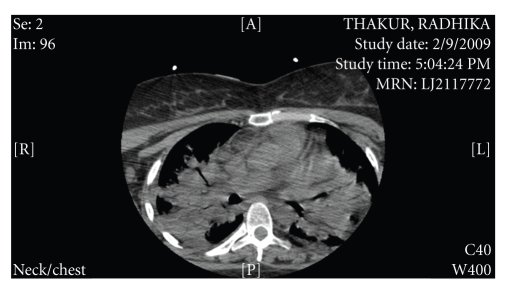
CT chest showing the diffuse bilateral pulmonary infiltrates.

CT chest (Figure 2) showed bilateral multilobar pneumonia. She had an elevated WBC count of around 12 000/mm^3^ and a platelet count of 129 000 at the time of admission. Her creatinine was 1.55 mg/dL. A metabolic acidosis (pH of 7.2) with bicarbonate measuring 13 mEq/L was noted. Thyroid function tests were consistent with sick euthyroid syndrome. The blood cultures grew *Streptococcus Pneumoniae* after 18 hours of incubation.

## 4. Clinical Progress

The patient was continued on Imipenem and Vancomycin in the ICU, and had significant clinical improvement. The *Streptococcus Pneumoniae* was sensitive to erythromycin, penicillin ceftriaxone, levofloxacin, and vancomycin. On day 4 in the ICU, the antibiotic coverage was de-escalated to Vancomycin. She was extubated on day 5 and transferred to floor. The swollen parotid gland regressed to normal size within 10 days. She subsequently became bradycardic on the day of anticipated discharge. Her EKG showed a sinus tachycardia and new ST wave changes. She did not have any shortness of breath or chest pain at that time. No new murmurs or any other stigmata of endocarditis were noted. She underwent transesophageal echocardiogram which showed no evidence of any myocardial abscess or wall motion abnormality. She did not make any troponin or CK-MB. Eventually, her sinus bradycardia subsided on its own without the use of pacing or pharmacological agents, and the patient was discharged after 2 more days without any more events. She completed her 2-week course of intravenous antibiotics. 

She is being followed after discharge by cardiology, infectious disease, and rheumatology. Her parotid gland returned to normal size and all the signs of inflammation subsided. Serial EKG showed reversal of the ST wave changes found before, and she remained asymptomatic.

## 5. Discussion

Invasive Pneumococcal disease continues to be the common cause of mortality and morbidity in all age groups, despite the diligent use of Pneumococcal vaccine in the community [5, 6]. One out of every 3 to 4 individuals affected with pneumococcal pneumonia develop invasive pneumococcal disease, with the bacteria seeding via the blood stream into meninges, heart, joints, spine, and soft tissues [1]. The major risk factors [7] for developing invasive pneumococcal disease are advancing age, being a young child, those patients with chronic systemic disease, those with immunosuppressive conditions [8], and patients with indwelling hardware.

Viruses, especially the Mumps virus, used to be the most common cause of acute parotitis [9] in the prevaccine era. The most common pathogens associated with acute bacterial parotitis are *Staphylococcus aureus* and anaerobic bacteria [2]. The predominant anaerobes include Gram-negative bacilli (pigmented *Prevotella * and *Porphyromonas* spp.), *Fusobacterium* spp., *Peptostreptococcus * spp*., *and *Streptococcus* spp. (*S. pneumoniae*). Gram-negative bacilli (including *Escherichia coli*) have also been reported [10]. There are rare cases in literature in which the Pneumococci seed the parotid gland [3, 4] causing its enlargement. Such cases may mimic other causes of bacterial parotitis but it should be kept in mind that Pneumococci have the potential of seeding into the blood stream and causing fatal bacteremia.

A bacterial infection of the parotid gland usually follows patients who are dehydrated, elderly, have poor oral hygiene, in patients receiving intensive care, and in newborns [2]. It has rarely been described in patients who are HIV-positive or other immunodeficiency syndromes [11, 12]. Rarely, parotitis has been described in Lupus patients [13]. Acute bacterial seeding should be considered in a patient who has presents with signs of septic shock and swelling of parotid gland. The abscess might need emergent drainage if any fluctuance is obvious but might not yield much pus in case of Streptococcal infections because of its diffuse spreading nature.

## 6. Conclusion

Acute bacterial parotitis can initiate the seeding of pneumococci in the blood especially in case of immunocompromised individuals and also may be a presenting feature in an individual as a parotid swelling. Prompt use of imaging studies and blood cultures followed by intravenous antibiotics may be life saving in this regard.

## References

[1] Brook I (2003). Acute bacterial suppurative parotitis: microbiology and management. *The Journal of Craniofacial Surgery*.

[2] Sheppard DC, Chambers HF (1998). Suppurative parotitis. *Western Journal of Medicine*.

[3] Stellbrink H-J, Albrecht H, Greten H (1994). Pneumococcal parotitis and cervical lymph node abscesses in an HIV-infected patient. *Clinical Investigator*.

[4] Payne RT (1940). Pneumococcal parotitis. *British Medical Journal*.

[5] Gentile JH, Sparo MD, Mercapide ME, Luna CM (2003). Adult bacteremic pneumococcal pneumonia acquired in the community. A prospective study on 101 patients. *Medicina*.

[6] Laupland KB, Gregson DB, Zygun DA, Doig CJ, Mortis G, Church DL (2004). Severe bloodstream infections: a population-based assessment. *Critical Care Medicine*.

[7] Lipsky BA, Boyko EJ, Inui TS (1986). Risk factors for acquiring pneumococcal infections. *Archives of Internal Medicine*.

[8] Nuorti JP, Butler JC, Gelling L, Kool JL, Reingold AL, Vugia DJ (2000). Epidemiologic relation between HIV and invasive pneumococcal disease in San Francisco County, California. *Annals of Internal Medicine*.

[9] Pickering LK, American Academy of Pediatrics (2003). Mumps. *Red Book: 2003 Report of the Committee on Infectious Diseases*.

[10] Pino Rivero V, Pantoja Hernández CG, González Palomino A (2006). Suppurative bacterial acute parotitis in adults. Clinic and microbiological findings in 10 admitted patients. *Anales Otorrinolaringológicos Ibero-Americanos*.

[11] Blanche P, Sicard D (1996). Pneumococcal parotitis in human immunodeficiency virus infection. *La Revue de Médecine Interne*.

[12] Stellbrink H-J, Albrecht H, Greten H (1994). Pneumococcal parotitis and cervical lymph node abscesses in an HIV-infected patient. *Clinical Investigator*.

[13] Azarisman SM, Heselynn H (2007). Systemic lupus erythematosus presenting as parotitis and secondary Sjogren's syndrome. *Singapore Medical Journal*.

